# Unusual Elongated Left Lobe of the Liver: A Cadaveric Case Report

**DOI:** 10.7759/cureus.110273

**Published:** 2026-06-04

**Authors:** Rajani Singh, Kavita Singh

**Affiliations:** 1 Anatomy, Graphic Era Institute of Medical Sciences, Dehradun, IND; 2 Physiology, Graphic Era Institute of Medical Sciences, Dehradun, IND

**Keywords:** abdominal cavity, diaphragm, hepatic tissue, largest, liver, spleen

## Abstract

An elongated left lobe of the liver is a prolongation of normal hepatic tissue reaching the left quadrant, sometimes encircling the spleen. Usually, it is asymptomatic, but in trauma to the upper left abdomen, this part of the liver is likely to get injured, mimicking splenic injury, as liver tissue and spleen possess the same echogenicity and density on sonography and computed tomography. In addition, it may be mistaken for mass or blood collection on imagery, leading to misdiagnosis and mismanagement. We present a case of an elongated left lobe of the liver touching the spleen and then passing upward obliquely, reaching the diaphragm. This anomaly was observed in a male cadaver of 60 years during routine teaching of the abdominal cavity for undergraduate medical students. The information will be useful in avoiding misunderstanding during the reading of ultrasound and computed tomography for abdominal traumas in the left hypochondrium.

## Introduction

The liver (Lr), the largest gland of the digestive system, comprises two lobes, namely, right and left, which are separated by the falciform ligament. The right lobe is restricted to the right hypochondrium, and the left lobe is enshrined in the left hypochondrium. Typically, the left lobe of the Lr (LeL) is related to the fundus of the stomach [1]. Very often, the Lr is subjected to various kinds of variations, such as variations in relation to fissures, underdevelopment or overdevelopment of the lobes of the Lr, and the existence of multiple lobes of the Lr [2]. Unduly large length of the LeL, also known as an elongated lobe or beaver’s tail Lr, though well documented in literature, is a rare variant. This aberrant anomaly of the LeL is detected incidentally and may present with abdominal symptoms like epigastric pain [3]. In the present case report, we document a case with variant LeL, which has been scarcely reported in the existing literature.

## Case presentation

**Figure 1:**
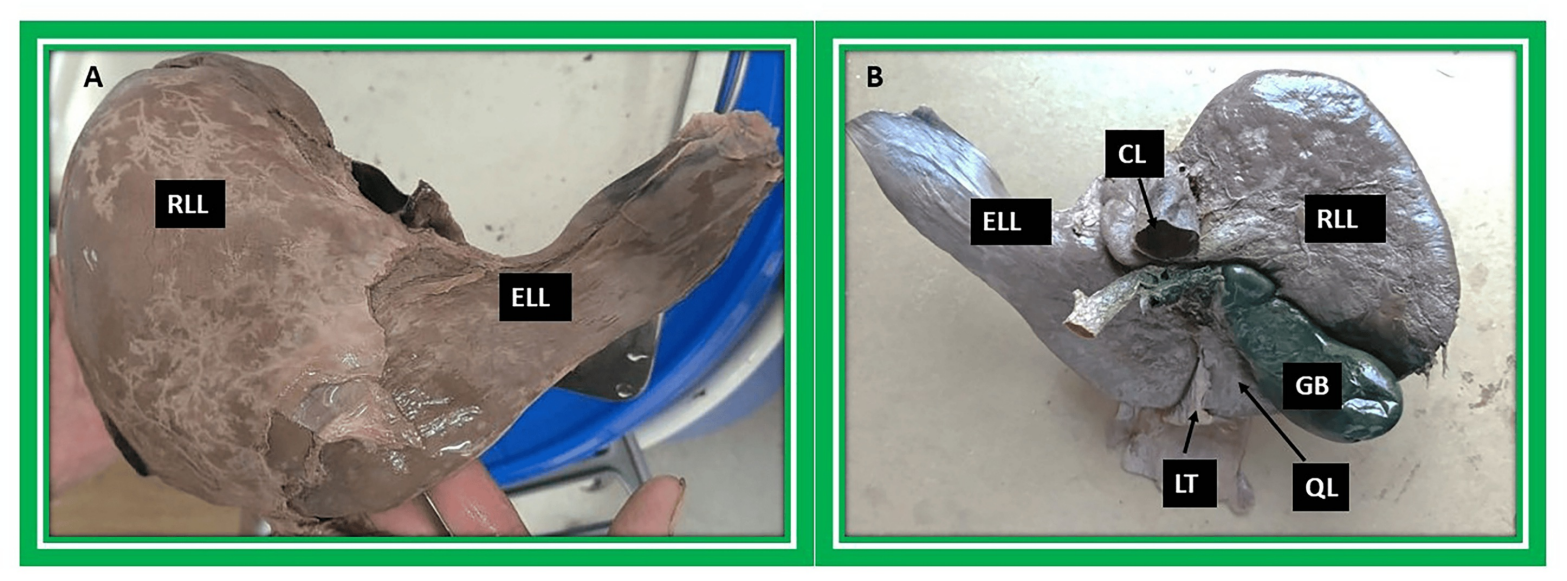
Elongated left lobe of the liver. (A) Anterior surface. (B) Visceral surface. RLL: right lobe of the liver; ELL: elongated left lobe of the liver; GB: gall bladder; LT: ligamentum teres; CL: caudate lobe; QL: quadrate lobe

During routine teaching of undergraduate medical students, the Lr was obtained from the visceral repository of the Dissection Hall of the Department of Anatomy. The authors noted elongated LeL (Figure 1) in a formalin-fixed male cadaver of 60 years. The anterior surface of this specimen was normal (Figure 1A). On the visceral surface of this Lr, the quadrate lobe and caudate lobe were normal in shape and orientation (Figure 1B). The fissure for the ligamentum venosum was very prominent. There was no other abdominal anomaly.

The study has been approved by the institutional review board of the institute vide number: GEIMS/IRB/2026/6:Cycle7.

## Discussion

The Lr is a main source of red blood cell formation in early fetal life, but later on, this function is taken over by the spleen and bone marrow. Like any other organ in the human body, the Lr is also subject to various congenital anomalies. Anomalies of the Lr, particularly with reference to its lobes, like multiple lobes or ectopic lobes, are scarce [4]. Extra lobes of the Lr may be due to heredity or may be influenced by environmental factors. One example of an additional lobe is Riedel's lobe, which remains connected to the main tissue of the Lr [5]. However, undue enlargement of lobes of the Lr, be it right or left, may lead to the squeezing of structures lying in the proximity or may hinder the normal function of the Lr [6].

The case presented presently is an example of excessive enlargement of LeL and is designated various names like beaver’s tail Lr and sliver of Lr. The elongated left lobe of the Lr is given the name beaver’s tail Lr as it is akin to a beaver's tail [7]. In rare situations, certain authors observed the beaver’s tail Lr surrounding the spleen [4,8,9]. As per these investigators, such an anomaly is difficult to discriminate from the spleen and the Lr in radiographs, leading to misinterpretation. In addition, this variation can be mistaken for trauma to the spleen or hematoma [4,8-10].

But in the present case, the elongated LeL does not surround the spleen but only touches the spleen, producing a kissing sign. This sign may be linked with excessive enlargement of the Lr and spleen along with other pathological conditions [11]. Contrary to this view, this sign may also be present in young lean but healthy people with no pathology. In the present case, there was also no pathology associated with this sign. One additional feature of the present study is that the elongated LeL passes obliquely upward, touching the diaphragm. This is a rare finding. Such anatomical variation may be mistaken for pathology, more so in an emergency, as in the case of performing an ultrasound to assess the ailment in case of trauma, specifically trauma to the left quadrant [12], along with planning of interventional procedures involving the abdomen [6]. The presence of an anomaly described in the present case warrants the attention of the clinicians, including radiologists, as the anomaly may be mistaken for splenic injury due to analogous densities and echogenicity on sonography and computed tomography [7].

Embryological basis of the present anomaly

The development of the Lr commences as a ventral bud at the meeting point of the foregut and midgut. The two lobes of the Lr are formed from this bud. During the 12th week of gestation, the two lobes are of equal size. But after this period, the hematopoietic function of the Lr is overtaken by the spleen and bone marrow, resulting in the regression of the LeL. Incomplete regression may be the cause of this anomaly [3].

## Conclusions

The elongated LeL, also known as the beaver’s tail Lr, is a prolongation of LeL consisting of normal hepatic tissue. This lobe either makes contact with or encircles the splenic tissue. This anomaly is so rare that only case reports are available describing it. In our case, the elongated lobe was in contact with the diaphragm, a very rare finding. Additionally, although this variation is more commonly reported in women, it was observed in a male cadaver. This variation needs the attention of clinicians, specifically those dealing with emergency cases, as in injury of the upper abdomen, it may be mistaken for injury to the spleen or a collection of blood, resulting in misdiagnosis and mismanagement, augmenting the risk of mortality. In case of trauma to the upper abdomen, a colored Doppler ultrasound is recommended in the literature so as to minimize the chances of misdiagnosis.
